# Impaired Topological Properties of Gray Matter Structural Covariance Network in Epilepsy Children With Generalized Tonic–Clonic Seizures: A Graph Theoretical Analysis

**DOI:** 10.3389/fneur.2020.00253

**Published:** 2020-04-16

**Authors:** Yongxin Li, Ya Wang, Yanfang Wang, Huirong Wang, Ding Li, Qian Chen, Wenhua Huang

**Affiliations:** ^1^Formula-Pattern Research Center, School of Traditional Chinese Medicine, Jinan University, Guangzhou, China; ^2^Guangdong Provincial Key Laboratory of Medical Biomechanics, School of Basic Medical Sciences, Southern Medical University, Guangzhou, China; ^3^Electromechanic Engineering College, Guangdong Engineering Polytechnic, Guangzhou, China; ^4^Department of Pediatric Neurosurgery, Shenzhen Children's Hospital, Shenzhen, China

**Keywords:** generalized tonic–clonic seizures, epilepsy children, gray matter volume, structural covariance network, graph theory, small-world

## Abstract

Modern network science has provided exciting new opportunities for understanding the human brain as a complex network of interacting regions. The improved knowledge of human brain network architecture has made it possible for clinicians to detect the network changes in neurological diseases. Generalized tonic–clonic seizure (GTCS) is a subtype of epilepsy characterized by generalized spike-wave discharge involving the bilateral hemispheres during seizure. Network researches in adults with GTCS exhibited that GTCS can be conceptualized as a network disorder. However, the overall organization of the brain structural covariance network in children with GTCS remains largely unclear. Here, we used a graph theory method to assess the gray matter structural covariance network organization of 14 pediatric patients diagnosed with GTCS and 29 healthy control children. The group differences in regional and global topological properties were investigated. Results revealed significant changes in nodal betweenness locating in brain regions known to be abnormal in GTCS (the right thalamus, bilateral temporal pole, and some regions of default mode network). The network hub analysis results were in accordance with the regional betweenness, which presented a disrupted regional topology of structural covariance network in children with GTCS. To our knowledge, the present study is the first work reporting the changes of structural topological properties in children with GTCS. The findings contribute new insights into the understanding of the neural mechanisms underlying GTCS and highlight critical regions for future neuroimaging research in children with GTCS.

## Introduction

Generalized tonic–clonic seizure (GTCS) is a subtype of generalized seizure that produces bilateral, convulsive tonic and clonic muscle contractions. People with GTCS showed significant emotional and behavioral problems, such as emotion, attention, language, and memory dysfunctions (1). The disorder is characterized by a disturbance in the functions of both hemispheres, which is caused by the electrical signals inappropriately spreading through the whole brain. Recently, through advances in both neuroimaging technology and analysis method of data, researchers have begun to detect the underlying neural mechanism of the disease. The behavioral abnormalities in epilepsy patients were suggested to be induced by the widespread neurobiological abnormalities in brains with GTCS in neuroimaging studies (2–5). In adults with GTCS, a functional reorganization of the dorsal attention network and default mode network (DMN) was observed (2). Additionally, a significant reduction of gray matter (GM) volume and corresponding behavior-neuroimaging correlation in the medial temporal part were detected in adults with GTCS (4). In children with GTCS, we also discovered significant changes of GM volume and brain activity in DMN, hippocampus, temporal, thalamus, and other deep nuclei in a recent multimodal magnetic resonance imaging (MRI) study (6). Although the epilepsy-related brain activity and anatomy changes in patients with GTCS were discovered in the neuroimaging studies, the whole-brain GM structural topology remains poorly understood.

As we know, the human brain contains billions of neurons, which connect with each other by synapses (7). Thus, the human brain can be considered as a complex network that enables highly efficient information communication (8). Graph theory is a powerful and comprehensive method for modeling the human brain as a complex network (9). The method can be used to detect the global and local topological properties of complex functional and structural network in neuroimaging domain. Recently, the method has been widely applied to investigate the human brain networks in healthy and neurological diseased populations (10–13). Using the graph theory method, researchers have discovered the topological characteristics in the normal human brain networks, including the small-world organization characterized by high clustering coefficients and short average path lengths (10, 14). In clinical disorder domain, researchers also detected and understood the cognitive impairment of the populations with neurological disorders via the brain's topological changes (13, 15). In patients with epilepsy, significant changes of the brain topological organization comparing with the normal controls have been discovered (12, 13). Moreover, adults with GTCS showed altered functional integration within DMN and disrupted functional and structural rich club organization of the brain network (16, 17). The nodal characteristic in the subcortical regions, temporal lobe and DMN were altered and the functional–structural coupling of brain network were changed in adult with generalized epilepsy (18, 19). Thus, analyzing the brain network topology in clinical disabilities based on the graph theory method could provide a potential method to understand the underlying neural mechanism. However, the recruited subjects were limited to adults and mainly focused on the disturbances in functional networks of GTCS in previous studies. The structural covariance patterns of gray matter volume in the GTCS children remain unclear and need to be investigated.

In one of our recent studies, we discovered that children with GTCS had significant changes of GM volume and functional activity in some regions (6). The result was not fully consistent with the discoveries in adults with GTCS (5, 17), implying a different GTCS effect on neuroimaging expressions of the brain between children and adults with epilepsy. In the present study, we aimed to investigate the topological properties of whole-brain structural covariance networks in children with GTCS through applying the graph theory method on the T1-weighted images. Based on the previous findings, we assumed that the brain structural covariance networks in both GTCS and healthy children would follow a small-world organization. We also hypothesized that children with GTCS may have a change in regional topological organization of GM structural covariance networks, involving the thalamus, DMN, hippocampus, temporal, and other deep nuclei that may relate to the GTCS children revealed in our previous study (6).

## Methods

### Subjects

The T1-weighted images reported in the present study were obtained from our previous research (6). Fourteen children with GTCS (four females, mean age: 54.36 ± 38.93 months) were collected in this study. The demographic and clinical information of all patients were listed in Table 1. The disease was diagnosed based on the detailed history and video-EEG telemetry result. The International League Against Epilepsy (ILAE) criterion was the basic guideline of the clinician for epilepsy diagnoses and classification. The inclusion criteria were as follows: (1) typical clinical symptoms of GTCS, such as tic of limbs followed by a clonic phase of rhythmic jerking of the extremities, loss of consciousness during seizures, and no partial seizures; (2) a specific pattern of electrophysiological activity measured by EEG in which generalized spike-and-wave or poly-spike-wave discharges were recorded; (3) no focal abnormality in routine structural MRI examinations. All patients were treated with at least one antiepileptic drug to control seizures before the recruitment (see Table 1). The used anti-epileptic drugs of the patients include topiramate, valproic acid, oxcarbazepine, and/or levetiracetam. All patients were seizure-free during the MRI examination processing. Twenty-nine healthy controls (17 female, mean age: 61.28 ± 26.66 months) were included without history of psychiatric illnesses or neurologic disorders. As some of the participants were too young to keep still during the scanning, the participants under the age of 4 years old were sedated (10% chloral hydrate) during the MRI scanning to reduce the body movement.

**Table 1:** Summary of the clinical characteristics of child epilepsy patients.

**Patient no**.	**Sex**	**Age**	**Age of epilepsy onset in months**	**Duration (months)**	**Antiepileptic drugs**
1	M	8	0.5	7.5	Oxcarbazepine, Valproic acid
2	M	140	104	36	Topiramate, Lamotrigine
3	F	55	2	54	Oxcarbazepine, Levetiracetam
4	F	55	19	36	Oxcarbazepine, Levetiracetam
5	M	80	6	74	Valproic acid, Levetiracetam
6	M	120	72	48	Topiramate, Levetiracetam
7	F	9	9	0.7	Valproic acid
8	M	68	68	0.3	Lamotrigine
9	M	40	18	22	Lamotrigine, Valproic acid
10	M	7	1	5	Oxcarbazepine
11	F	39	4	36	Topiramate, Lamotrigine
12	M	52	9	41	Topiramate, Levetiracetam
13	M	37	36	2	Valproic acid
14	M	51	9	42	Lamotrigine

*F, female; M, male*.

Written informed consent was obtained from the parents or guardians of all participants prior to the data acquisition. The present study was carried out according to the approved guidelines and in accordance with the Declaration of Helsinki. All methods used were approved and monitored by the Medical Research Ethics Committee of the Shenzhen Children's Hospital.

### MRI Acquisition and Preprocessing

**Figure 1:**
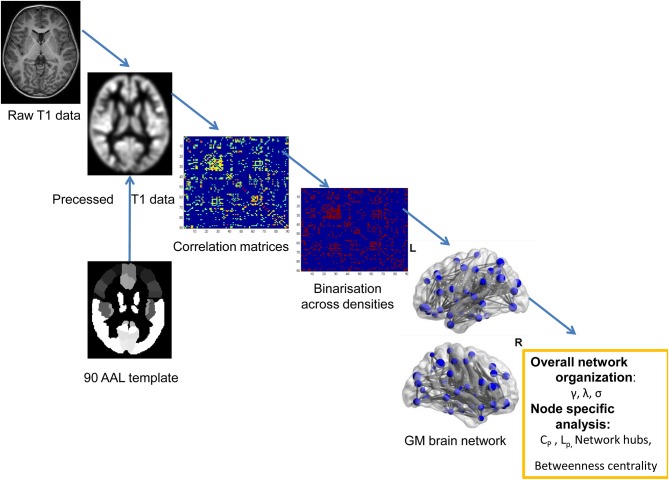
The flowchart for constructing the GM structural network using T1-weighted images.

MRI scanning was performed using a 3T Siemens TrioTim scanner (Germany, 8-channel birdcage head coil) at the Department of Radiology, Shenzhen Children's Hospital. During the scanning, each patient lay at the supine position with the head fixed by foam cushions. The participants were asked to keep awake and relax with his/her eyes closed. The structural MRI data were obtained using a 3D-MPRAGE sequence: 160 sagittal slices, TR = 2,300 ms, TE = 2.26 ms, flip angle = 8°, FOV = 200 × 256 mm^2^, thickness = 1 mm. A graph theory method was used to detect the topological organization of GM structural covariance network (see the flowchart of Figure 1).

Structural MRI scans were preprocessed using the CAT12 (http://dbm.neuro.uni-jena.de/cat/) based on SPM12 (http://www.fil.ion.ucl.ac.uk/spm). Correction of bias-field inhomogeneities were performed on all T1-weighted images and then the corrected structural data were segmented into the GM, white matter (WM), and cerebrospinal fluid (CSF). Afterwards, the DARTEL algorithm was adopted to normalize the data spatially using an affine transformation (20) and a customized DARTEL template was produced by the GM and WM segments data of all subjects. The created customized template was registered to the ICBM template in the Montreal Neurological Institute (MNI) space in the CAT12 Toolbox. All of the structural images were re-analyzed by using the customed DARTEL template to obtain normalized and modulated tissue probability map of GM image. The modulated GM was written with an isotropic voxel resolution of 1.5 mm. Visual checks for artifacts were performed on the preprocessing data. Outliers were identified by the sample homogeneity module and defined as two or more standard deviations outside of the GM volume sample distributions center. No participant was excluded by the automated quality check protocol.

### GM Structural Covariance Networks Construction

The extracted GM volume maps were used as the input to a graph-analysis toolbox (GAT) to construct the GM structural correlation networks (21). The Matlab package can be used to detect the inter-group differences in the brain network topology. We employed the AAL template to assign the brain into 90 cortical and subcortical regions of interest (ROI) (22). Regional GM volumes of each ROI were extracted and corrected of age and gender. Pearson correlations between the regional GM volumes were performed across subjects to generate a 90 × 90 association matrix for each group. Adjacency association matrices were binarized and derived at a range of densities (0.15–0.5, with an interval of 0.01). Inter-group differences of network topologies were compared across the range.

### Global and Regional Network Analyses

To describe the topological organization of GM structural covariance networks, intra-group and inter-group differences in small-world parameters were analyzed (23, 24). The human brain can be regarded as a small-world network that has the highest clustering coefficient (C_p_) and shortest path length (L_p_). The C_p_ of a node is defined as the number of edges that exist between its neighbors. The C_p_ of a network can be calculated by the average of C_p_ across nodes, which can reflect the network segregation of the brain. The L_p_ is defined as the shortest average path length between any two nodes. The normalized clustering coefficient (γ) and normalized path length (λ) were calculated, respectively, by comparing the C_P_ and L_p_ to the mean C_p_ and mean L_p_ of 1,000 random network (25). A network's small-world index is defined as σ = γ/λ (26). The index σ can reflect the balance between segregation and integration among all nodes of the network. In the present study, small-world characteristics were calculated at the minimum connection density (*D*_min_ = 0.15) as well as across a range of densities (0.15–0.5, increment of 0.01) using the Area Under the Curve (AUC). Global network measure curves were calculated and compared the network topologies between groups across the range of network densities (21). A connectome was considered to be small-world when the characteristic path length is comparable to that of a random network and the clustering coefficient is significantly higher than that of a random network.

In the present study, the nodal characteristics of the GM structural covariance network were examined and the differences of regional network between groups were analyzed. Nodal betweenness centrality is an important index which is defined as the fraction of shortest paths passing through a node (24). The graph index is used to detect important functional or anatomical connections. The quantified nodal betweenness centrality was normalized by the mean network betweenness centrality. Inter-group differences of the normalized nodal betweenness centrality were compared (27, 28).

### Network Hubs

To investigate the strength and density of the total network, hubs were also detected based on the entire sample. Hubs are the most globally connected regions in the brain and are essential for coordinating brain function through the connectivity with numerous brain regions. Hubs play a central role in integrating diverse information sources and supporting fast information communication with minimal energy cost. The criteria for defining hub is that the node's betweenness was at least 1 standard deviation higher than the mean network betweenness (21).

### Comparing Network Metrics Between the Groups

Inter-group differences in global and regional network metrics were analyzed with a non-parametric permutation approach (1,000 permutations) (28, 29). For each permutation, the GM volume metrics of all participants were randomly reassigned into two new groups. An association matrix for each randomized group was obtained. The adjacency matrices were binarized and then estimated by thresholding at the range of 0.15–0.5. The inter-group differences of the randomized groups were calculated at each network density. The actual inter-group network difference was analyzed in the corresponding permutation distribution, and the corresponding *p*-values were computed based on the percentile positions. Brain Connectivity Toolbox was used to quantify the network metrics (24) and GAT was applied to detect the structural covariance network differences between the groups (21). BrainNet Viewer was used for network visualization (30).

Inter-group differences in regional network metrics were investigated, which included the nodal betweenness difference at *D*_min_ threshold. We also generated the 95% confidence interval for each metric to see if the observed inter-group differences are statistically significant or not (*p* < 0.05, uncorrected).

## Results

### Intra-Group Global Network Metrics

**Figure 2:**
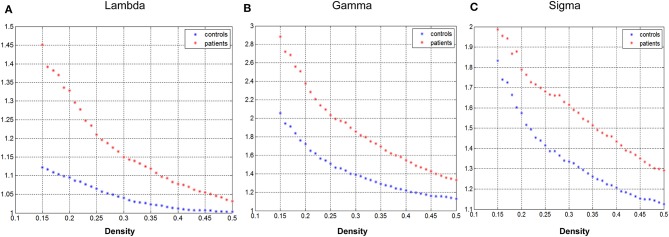
Changes in global network measures as a function of network density. Normalized path length (**A**, Lambda), normalized clustering coefficient (**B**, Gamma), and small-world index (**C**, Sigma) of the GTCS child and healthy control network.

Figure 2 demonstrated that changes in global network play a function of network densities. Both groups' network followed a small-world organization across the densities from 0.15 to 0.5 (γ >>1, λ~1, σ = γ/λ >>1).

### Inter-Group Differences in Global Network Metrics

**Figure 3:**
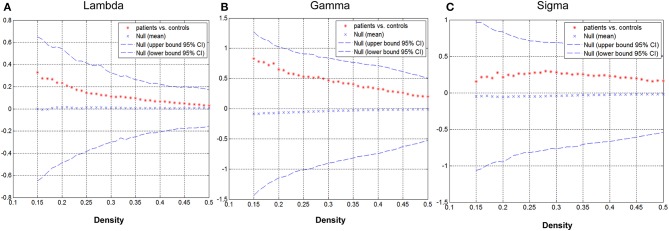
Differences between GTCS and healthy control participants in global network measures as a function of network density. The 95% confidence intervals (CIs) and group differences in normalized path length **(A)**, normalized clustering coefficient **(B)**, and small-world index **(C)**. The positive values show patients > controls and negative values show controls > patients.

Group differences in global network metrics were examined at a range of densities (0.15–0.5). No significant inter-group differences were detected for all small-world parameters (Figure 3). The AUC for global network measure curves were also compared between groups. The network of children with GTCS had not significantly changed AUC for all small-world parameters compared with the normal control network: γ (*p* = 0.129), λ (*p* = 0.168), σ (*p* = 0.217).

### Inter-Group Differences in Regional Network Metrics

**Figure 4:**
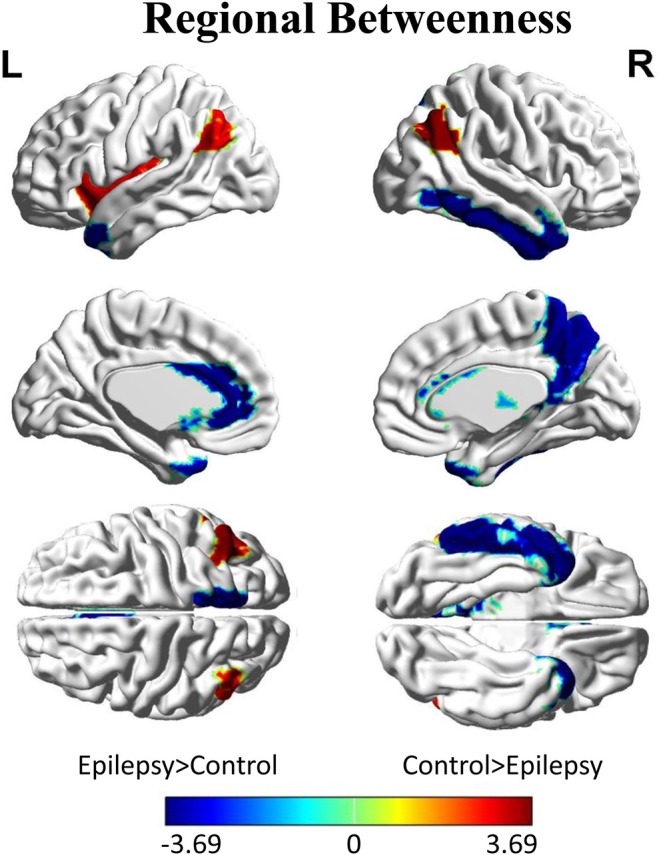
Differences between GTCS and healthy control participants in regional betweenness. Regions that showed significant differences between both groups in regional network topology were presented at minimum density of full connectivity mapped on ICBM152 surface template. The color bar represents log(1/*p*-value). The hot colors in the color bar represent regions that have significantly higher nodal betweenness in the healthy controls than in the GTCS children, while cold color denote regions with significantly higher nodal betweenness in the GTCS children than in the healthy controls. L, left; R, right.

Inter-group differences in regional network metrics of nodal betweenness centrality were shown in Figure 4. Regions including the left insula and bilateral angular demonstrated significant decrease of nodal betweenness centrality in children with GTCS. Conversely, some regions, including the bilateral temporal pole of middle temporal gyrus (MTP), left caudate, left anterior cingulum gyrus (ACC), right thalamus, right precuneus, and inferior temporal gyrus (ITG), showed a significant increase of nodal betweenness centrality in children with GTCS. None of these regions survived after correcting for multiple comparisons (*p* < 0.05).

### Network Hubs

**Figure 5:**
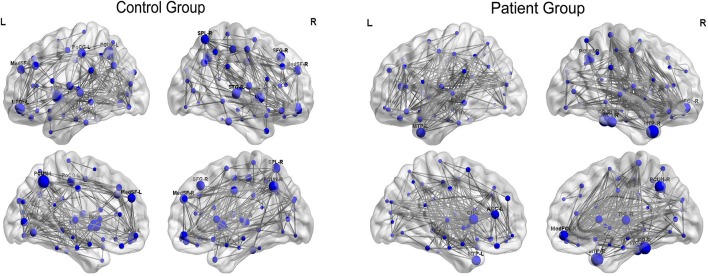
Constructed networks and corresponding hubs for both groups. The volume of the spheres represents the betweenness centrality of the corresponding brain region. MedSF, medial superior frontal gyrus; SFG, superior frontal gyrus; INS, insula; MFG, middle frontal gyrus; MedFOr, orbital medial frontal gyrus; PoCG, post-central gyrus; PCUN, precuneus; SPL, superior parietal lobule; STG, superior temporal gyrus; MTP, temporal pole of middle temporal gyrus; CN, caudate; THL, thalamus; CN, cuneus; FG, fusiform gyrus; ITG, inferior temporal gyrus; L, left; R, right.

Figure 5 displayed the hub network layouts mapped on an ICBM152 surface template for the normal and patient group. Hubs determined for the control group network included the bilateral medial superior frontal gyrus (MedSF), bilateral insula, bilateral precuneus, left orbital superior frontal gyrus (SFOr), left post-central, right orbital medial and middle frontal gyrus, right superior parietal lobe, and superior temporal gyrus. Hubs for the patient network included the bilateral caudate, bilateral MTP, left anterior cingulum, right orbital medial frontal gyrus (MedFOr), right fusiform, right precuneus, right thalamus, and ITG.

**Figure 6:**
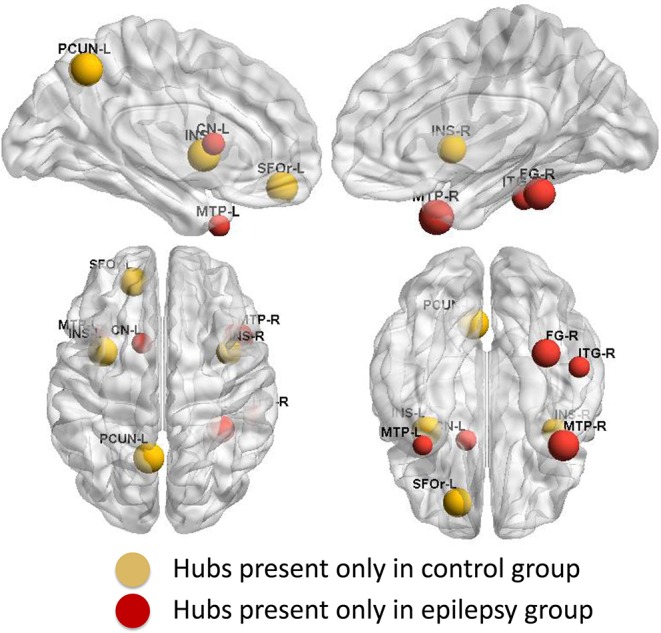
Group-specific hubs. Yellow color highlights hubs specific to healthy controls' network and red color represents hubs specific to GTCS children's network.

Group-specific hubs are shown in Figure 6. Right precuneus and right MedFOr were two common network hubs in both groups. Patient-only hubs included the bilateral MTP, left caudate, right fusiform, and ITG. On the contrary, the bilateral insula, left precuneus, and SFOr were the specific hubs only presented in the normal controls.

## Discussion

In the present study, graph analyses were used to investigate the differences in GM structural covariance networks between children with GTCS and healthy controls. Although the GM structural covariance networks in the patient group followed a small-world organization across a range of densities similar to the control group's network, significant alterations of the topological property were found in the GM structural covariance network in patient group. Specifically, epilepsy children were characterized by significantly increased centrality of structures including the bilateral MTP, left caudate, left ACC, right thalamus, right precuneus, and ITG. Significant alterations in the regional topological properties with reduced centrality were found in the regions including bilateral angular and left insula. The results revealed that the GM structural covariance network's small-world property was changed in children with GTCS. The observation confirmed the hypothesis and suggested a widespread neurobiological injury in children with GTCS. To our knowledge, the present study is the first research reporting the alteration of GM structural topology properties in children with GTCS.

### Global Network Measures

The GM structural covariance network of the normal control group followed a small-world organization across the range of densities (Figure 2). The results are consistent with the findings in a previous study that normal human brain is an architecture with simultaneous high segregation and integration (14). In children with GTCS, the structural network also followed a small-world organization, indicating that the architecture of brain in the GTCS children may be balanced between the local and global information processing. The small-worldness property of the brain GM structural covariance network has been proven via T1-weighted MRI in both healthy individuals (31–33) and epilepsy patients (12, 17, 34). In the present study, we did not find significant inter-group differences in the global network properties of the GM network. The result is differentiated from the findings in adults with GTCS in the previous neuroimaging study, in which the GTCS adults demonstrated a decreased small-world topology and normalized clustering coefficient (18). The possible explanation for the inconsistency may be the different development stage of the brain between the adult and children participants. In children with neural system diseases, the brain is still under development and tends to have an organization influenced by learning new skills, experience, and the neural system disease (35–37). As a result, though the brain structure of children with GTCS could be influenced by the epilepsy, the development of neurons could reduce the disease effect and minimize the topological changes. On the contrary, in adults with GTCS, the brain is fully developed and the structural reorganization may only result from the epilepsy effect. Due to the effect of both brain maturity and disease, the global GM network topology properties in the GTCS children did not show significant changes as in the GTCS adults compared with the normal controls. The above discussion indicated different properties of the global GM network properties existing between children and adults with GTCS.

### Regional Network Measures

GTCS is a neurological disorder. Patients with GTCS demonstrated significant changes of the brain GM volume and activity in a specific regional network (4, 6). In the present study, differences in nodal betweenness centrality were tested between the epilepsy children and healthy controls. Bilateral angular gyrus and left insula of children with GTCS showed significant lower betweenness centrality. A node with high structural betweenness centrality indicates that the node is highly interactive with the other nodes (31). Significant decrease of betweenness centrality in angular gyrus and insula of our results may be induced by the epilepsy disruption of the structural pathways. So, the interaction with the other nodes of bilateral angular gyrus and left insula was reduced in children with GTCS. A number of epilepsy studies have shown the GM volume decrease in insula of patients with GTCS (6, 38) and juvenile myoclonic epilepsy (39). The structural impairment of the insula would change the related pathways connected with the insula and affect the motor and somatosensory function. Angular gyrus is the region known to be involved in the complex cognitive functions, such as language and sensory information integration. Left angular belongs to the DMN, while in adults with GTCS, the DMN showed abnormal connectivity and reduced functional integration (5, 16). The decreased centrality of left angular was consistent with these previous studies of functional connectivity changes in angular, which indicated the abnormal role of the angular in information transport and integration (40). The potential participation of functional interactions in angular gyrus was decreased in children with epilepsy. The significant decrease of nodal betweenness centrality in angular gyrus and insula may be the neuroimaging expression for the damage of the cognitive function in GTCS (1, 41). The changed region centrality in the relevant regions may lead to decreased cognitive function of children with GTCS.

In the present study, we detected a significantly increased betweenness centrality in the right thalamus region. The result was in line with the above neuroimaging studies in GTCS of both adults and children (6, 38, 42, 43). Thalamus is a core region that plays an important role in the transmission of epileptic activity via cortical–thalamic–subcortical circuits (44, 45). Seizure can induce brain structural and functional damage in the thalamus regions of participants with epilepsy (46–49). In adults with GTCS, the GM volume of thalamus decreased significantly while the activation and functional connectivity of thalamus to other regions changed significantly (38, 42, 43). In children with GTCS, a significant decrease of GM volume and increase of brain activation in bilateral thalamus had also been detected in a recent study (6). The alterations could be resulted from the abnormal cortical–subcortical electrical discharges transferring through the thalamus and reflect the co-occurrence between the tonic seizure activity and cognitive impairment. Based on the previous findings, the changes of thalamus can be considered as a common expression of the injurious effects of epileptic caution. Also, nodal betweenness centrality is an important index that can reflect the number of shortest paths passing through a node. The high betweenness centrality in thalamus we detected in the present study may indicate that the number of shortest paths passing through the thalamus was increased. Moreover, the abnormal cortical–subcortical electrical discharges were transferred through the thalamus. Thus, we can say that the thalamus acts as a bridge to connect the epilepsy-related regions. The constructed GM structural covariance network of thalamus in the present study revealed important anatomical cortical–thalamic–subcortical connections in GTCS children.

In addition to right thalamus, increased betweenness was also detected in bilateral MTP, left caudate, left ACC, right precuneus, and ITG of children with GTCS. The ACC, ITG, and precuneus belong to DMN and are related to multiple highly integrated functional systems. The result is consistent with the previous study that the connections between DMN and other regions were enhanced significantly in adult with GTCS (2). Combining with the findings in angular gyrus and insula, we can see that both increased and decreased betweenness of DMN regions were found in our study. The possible explanation may be the unbalanced resting-state networks activity in DMN of children with GTCS. Further studies are needed to confirm the view. The temporal pole is a core site considered as the seizure genesis within the temporal lobe seizure networks (50). The patients with temporal lobe epilepsy usually demonstrated significant abnormalities in temporal pole (51). For adults with GTCS, the interhemispheric functional connectivity between the bilateral temporal poles was weaker in patients than in normal controls (52). MRI-based morphometric correlation analysis revealed that the adult patients with GTCS had a less correlation between the thalamus and temporal pole (53). The brain connectivity pathway in bilateral temporal pole would be affected by seizure in adult. Children with GTCS of the present study showed significantly high betweenness in bilateral MTP structure network. The result is different from the adults with GTCS. Similar to the difference in global network properties between adults and children patients, the difference of betweenness may also count from the different brain development stage between children and adults. Additionally, the discovered high betweenness in the epilepsy-related regions (thalamus, temporal lobe) indicated that the regions may increase the interaction with other regions to compensate for the need of cognition function in children with GTCS.

In the present study, the bilateral significant changes of betweenness were only discovered in the angular and MTP, while in other hemispherical regions, unilateral significant changes of betweenness were detected. The reason for the lateralization effect may lie in the non-homogeneity of the epileptogenic focus location. By the compensation theory, the right hemisphere regions were possibly recruited to adapt the brain organization in children with GTCS. Hence, significant increase of betweenness in the right hemisphere regions was detected in the present study. The result was also proved by our recent study that shows that children with GTCS showed a significant correlation between the brain activity and epilepsy duration only in right thalamus (6). The phenomenon was supported by the network hub results in the present study, where some hubs were only found in the right hemisphere of the GTCS children who presented left hemispherical lesions. However, since we also detect both significant decreased and increased changes of betweenness in some left hemisphere regions but not in the homologous right hemisphere regions, the phenomenon cannot be completely explained by the compensation theory. Future studies should focus on the lateral effect of GTCS children with unilateral epileptogenic focus.

### Network Hubs

Both groups' networks showed a number of common hubs, such as the right precuneus and right MedFOr. All the common regions belong to the DMN and have been reported as pivotal nodes of human cortical network (40). The correlation between the structural and functional connection in the DMN was significant within the healthy participants (54, 55). The common hubs discovered in the DMN might indicate that the network hub properties in DMN can tolerate the effect of epilepsy. These previous studies on the functional and structural network correlation in the DMN were adults. The subjects of the present study were children. It is not clear whether the functional and structural covariance connection in the DMN of children has similar correlation as in adults. Thus, the above view of DMN retaining the network hub properties needs further investigation in the future. Also, the coupling of functional and structural connectivity networks has been found to increase with age (56). Adults with GTCS showed a disrupted functional connectivity related to DMN, which indicates that the information communication of DMN was influenced by GTCS (5). In the present study, the network hub properties of DMN were retained in children with GTCS. This result in some aspect showed that the neural mechanism of GTCS in children was different from the adults. The present results might provide meaningful information that different brain organization between children and adults with GTCS might lead to developmental changes of the brain.

Conversely, divergent distribution regions of network hubs between the epilepsy and healthy controls were also reported in the present study. The bilateral MTP, left caudate, right fusiform, and ITG were the highly GM structural covariance network hubs presenting only in the GTCS children. The network hub analysis results are consistent with the regional topology analysis findings. Most of the epilepsy specific hubs also showed a significant increase of the betweenness. The consistency between the network hubs and the betweenness may indicate an important role of the hubs in the interaction with other regions to meet the need of cognition function in children with GTCS.

On the contrary, the bilateral insula, left precuneus, and SFOr were the highly GM structural covariance network hubs presenting only in the normal controls. The results indicated that the GM structural covariance network hub property of bilateral insula, left precuneus, and SFOr was disrupted in children with GTCS. The results are in accordance with previous discoveries that patients with GTCS had aberrant core hub role of regions including precuneus, orbital frontal cortex, insula, and putamen (17, 18). Missing structural hubs in children with GTCS may reflect that epileptic actions can induce long-term injurious effects on the brain. In the present study, the changed network hubs in bilateral insula, left precuneus, and SFOr of GTCS children reflected a GM structural covariance network abnormality similar to the GTCS adults (17). Also, previous functional MRI studies on epilepsy have found that adults with idiopathic generalized epilepsy showed decreased functional connectivity of medial prefrontal cortex and precuneus (42, 43). The functional role of the prefrontal cortex and precuneus was disrupted after epilepsy. Combining with the above neuroimaging studies, our findings of loss hubs in children with GTCS tend to imply that abnormalities in the organization of GM structural covariance networks have important implication for neural function and cognitive decline observed in children with GTCS.

### Limitations

The present study has several limitations. First, the sample size of the patient group was relatively small. Future studies with large samples should be considered to provide further insights. Second, due to the nature of the T1 data focused in the study, we cannot estimate one graph per subject and failed to perform the correlation analysis between the epilepsy duration and network results. Third, we used the cross-sectional design in the present study. Longitudinal design is needed in the future to further repeat the results and assess whether the changes of network graph properties is the consequence of seizures. Fourth, the present study did not consider the medication effects on the topological properties of the T1 structural network. The sample size of patients was relatively small and the medicine used was not the same among subjects. This fact would have potential effects on the topological results. Finally, although the graph properties of GM structural covariance network were explored to understand the GTCS epilepsy effect in children, white matter structural information was not included in the present study. A previous study has found that the brain white matter functional or volume has shown physiological relevance (57). Future studies should consider the effect of this factor on brain network analysis.

## Conclusion

In summary, the present study using the graph theory method investigated topological properties of GM structural covariance network in children with GTCS. Both increased and decreased betweenness centrality were discovered in children with GTCS compared to normal controls. Significant changes of regional betweenness centrality within the GTCS group were mainly found in thalamus, temporal pole, and DMN that have been implicated in a previous GTCS study. The network hub analysis results were in accordance with the regional betweenness, identifying a disrupted regional topology of GM structural connectome in children with GTCS. To sum up, children with GTCS demonstrated specific changes of the network properties, which would provide meaningful information about brain organization led by brain development. The results highlight our understanding of the neural mechanism of GTCS in children and the effects of GM structural neural organization in GTCS.

## Data Availability Statement

The datasets analyzed in this article are not publicly available. Requests to access the datasets should be directed to chenqian68@126.com or yxin-li@163.com.

## Ethics Statement

The studies involving human participants were reviewed and approved by the Medical Research Ethics Committee of the Shenzhen Children's Hospital. Written informed consent to participate in this study was provided by the participants' legal guardian/next of kin.

## Author Contributions

YL, YanW, and YaW conceived and designed the experiments. YaW, QC, and YanW performed the experiments. YL and YaW analyzed the data. YL and DL contributed reagents, materials, and analysis tools. HW, QC, and WH responsible for patient management and conceptualized the study. YL wrote and revised the paper.

### Conflict of Interest

The authors declare that the research was conducted in the absence of any commercial or financial relationships that could be construed as a potential conflict of interest.
